# Investigation of the Impact of Load on the Magnetic Field Strength of the Crane by the Magnetic Metal Memory Technique

**DOI:** 10.3390/ma13235559

**Published:** 2020-12-06

**Authors:** Agnieszka Koson-Schab, Janusz Szpytko

**Affiliations:** Faculty of Mechanical Engineering and Robotics, AGH University of Science and Technology, A. Mickiewicza Av. 30, PL30-059 Krakow, Poland; szpytko@agh.edu.pl

**Keywords:** metal magnetic memory, magnetic field non-destructive evaluation, diagnostics, gantry crane

## Abstract

The paper deals with the problem of applicability of the metal magnetic memory (MMM) technique in the crane structural inspection and monitoring. The MMM method does not require the external magnetization of a structure that results in reduction of downtime of maintenance operations. Measurement of the intensity of the self-magnetic leakage signal can be an alternative to other non-destructive methods used for inspection of a large crane’s structure and equipment. However, the complexity of the residual magnetization effect in the MMM technique is the problem with its application. Thus, the magnetic flux leakage behavior on the crane girder surface under different measurements and the crane’s load conditions is analyzed based on the results obtained during experiments carried out on the overhead traveling crane.

## 1. Introduction

Currently, there is a growing interest in obtaining information from the research of operating structures in terms of their degradation processes for the purpose of improving their construction, manufacturing, and operation. Construction safety requirements are related to the development of new diagnostic methods enabling the detection and localization of structural degradation.

Structures of large-scale industrial transport devices, subjected to loads resulting from the type of their work, are exposed to high stressors. These stressors affect the fatigue strength of the structure as well as damage the material structure. In the interest of safety, you should perform frequent diagnostic tests of these devices.

Nowadays, technical diagnostics increasingly use non-invasive diagnostic methods for diagnosing or monitoring (continuous control) without interrupting the normal operation of the object [1,2,3]. One of the least invasive methods of diagnosis is magnetic metal memory (MMM). This is used for inspection of welds, structural elements such as loaded cyclically on rotors, gears, steel ropes, and much more. The MMM method is one of the fastest and cheapest for detecting and assessing early caused damage based on the natural stress-induced magnetic flux leakage (MFL) [4,5].

The MMM method, based on detecting the impact of changes in the residual magnetization of the examined object on its magnetic flux leakage, is increasingly used in diagnostics. It allows indicating places or dangerous areas, cracks, or other defects of elements made of ferromagnetic materials [6,7].

The MMM method is based on the magneto elasticity Villari effect. Local disturbances in the earth’s magnetic field are measured at the surface of ferromagnetic materials. Ferromagnetic materials in the presence of the geomagnetic field are subjected to stress, which causes the induction of a magnetic field [8,9,10,11].

The analysis of the test results consists of comparing the normal and tangential components of the magnetic flux leakage along the measuring path.

It is based on the known phenomenon of magnetic flux leakage of material with different magnetic permeability. Changes in the sign and leakage magnetic field *Hp* value are indications on the basis of which the assessment of stress and strain in the material of the tested element is made, define *Hp* by the Formula (1) [12]:

(1) $$H_{p}=\frac{1}{\mu_{0}}B_{p}$$

where:

*B*_0_ is the vertical value of the magnetic induction component [T];
*μ*_0_ is vacuum magnetic permeability 4π × 10^−7^ [H/m].

Areas of stress concentration are detected in which processes leading to a reduction in material strength can occur [11,13,14,15].

The MMM method can detect the residual magnetic field due to mechanical stress and geomagnetic field and can be performed without artificial magnetization. The magnetometer using the MMM method is based on magnetoresistance effects. It is characterized by a small volume, high sensitivity, and reliability. This makes it easier to detect the fatigue of the tested elements [2,16].

It is widely accepted that the magnetic state of ferromagnetic material may be irreversibly altered by mechanical loading due to magneto-elastic effects [17]. On the macroscopic scale, there are close relationships between the mechanical and magnetic properties of ferromagnetic material. Under the influence of mechanical stress in the ferromagnetic material, deformation occurs and changes the internal magnetic field. In general, the MMM is an after effect which is directly correlated with the distribution of natural magnetic fields and the accumulated stress-strain which has been experienced by the ferrous or paramagnetic material.

The heterogeneity of the structure, mechanical properties, and the magneto-plastic effect are the result of the leakage magnetic field, which will lead to an irreversible increase in the magnetic induction under a weak magnetic field. The ferromagnetic element is subjected to the external stresses in a certain direction [18].

The MMM signals are a weak magnetic signal, which consists of vulnerable factors such as lift-off height, chemical composition, the specimen shape, the notch shape, the heat-treating technology, the initial magnetization state of ferromagnetic materials, and environmental magnetic field [10,14,19,20].

The objective of the paper is to determine the experimental impact of the structure load change on the level of the own magnetic field, using the MMM method and measuring the magnetic field strength in the elastic deformation zone, for the purpose of inferring changes in the technical condition of the structure, on the example of the girder of the overhead crane.

## 2. Crane Girder Bridge Testing Approach with Use MMM Technique

The aim of the experiment was to determine the impact of the load change on the level of self-magnetic leakage field (SMLF)of a double girder bridge crane in terms of analyzing the possibility of using its residual magnetic flux.

As a result of stresses and plastic deformations, changes in the structure of the material caused by fatigue, local irregularities are observed as local changes in the degree of the material magnetization.

The value measured in the test is the value of the selected component of the magnetic field strength *H* measured near the diagnosed object, represented by Formula (2):

(2) $$H=\frac{B}{\mu_{r}\cdot \mu_{0}},$$

where [21]:

*H* is magnetic field strength [A/m],
*B* is magnetic induction [T],
*μ_r_* is relative magnetic permeability,
*μ*_0_ is vacuum magnetic permeability 4π × 10^−7^ [H/m].

**Figure 1:**
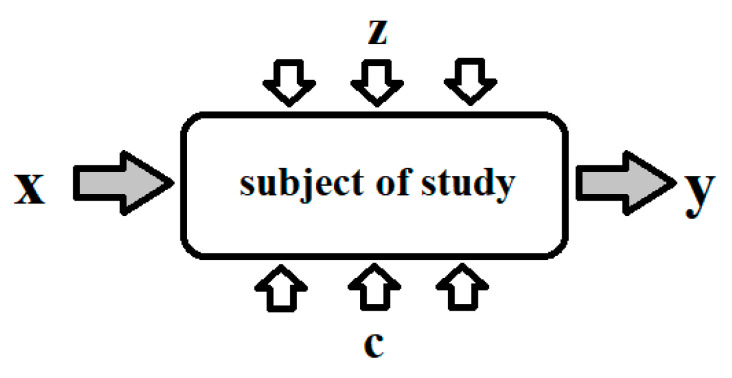
Block diagram of the research subject.

After analyzing the available literature on the research of objects using the MMM method, our own test plan was developed to reduce the cost and time of the experiment. The research plan cover: object model, the duration of individual experiments, identifying confounding factors and constants. Figure 1 shows the test object model.

The model research object (the girder of a double-girder overhead crane) can be represented by the function defined by Equation (3):

*y*(*t*) = *f* (*x, z, c, t*), (3)

where:

*y*—output values: magnetic field strength *H* in the tested girder section in [A/m];
*x*—input values: *x =* {*x*_1_*, x*_2_};
*x*_1_—beam load time in [s],
*x*_/1_—beam deflection in [mm],
*c*—solid factors: *c = {c*_/1_*, c*_/2_*, …c_i_}*;
*c*_1_—the high of raising in [mm], *c*_2_—load lifting speed in [m/s], *c*_3_—ambient temperature of crane in [°C], *c*_4_—crane girder load in [kg], *c*_5_—the place on the girder where the load was applied in [m], *c_i_*—other that did not affect the results of the tests carried out,
*z*—interference: *z =* {*z*_1_*, z*_2_*, …z_i_*};
*z*_1_—measurement errors, *z*_2_—magnetic field disturbances caused by the device’s operation in [A/m], *z*_3_—material structure, *z_i_*—factors unnoticed affecting the measurement.

The SMFL signal measured using the MMM method is influenced by static and dynamic loads, movements of driving and lifting mechanisms, and transient states of the drive systems.

The frequency inventers used in the crane drive system generate the external disturbances that affect the measurement signal. This drawback was eliminated by carrying out the measurement experiments when crane motors and frequency inverters were turned out. The other exogenous conditions, such as temperature (if the measurement is performed in the temperature range −20–60 °C), have minor impact on the measurement results.

**Figure 2:**
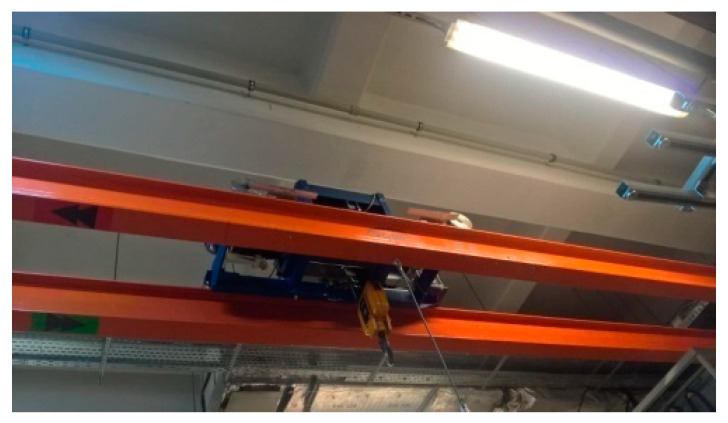
The tested double-girder overhead traveling crane with a trolley and a hoist.

**Figure 3:**
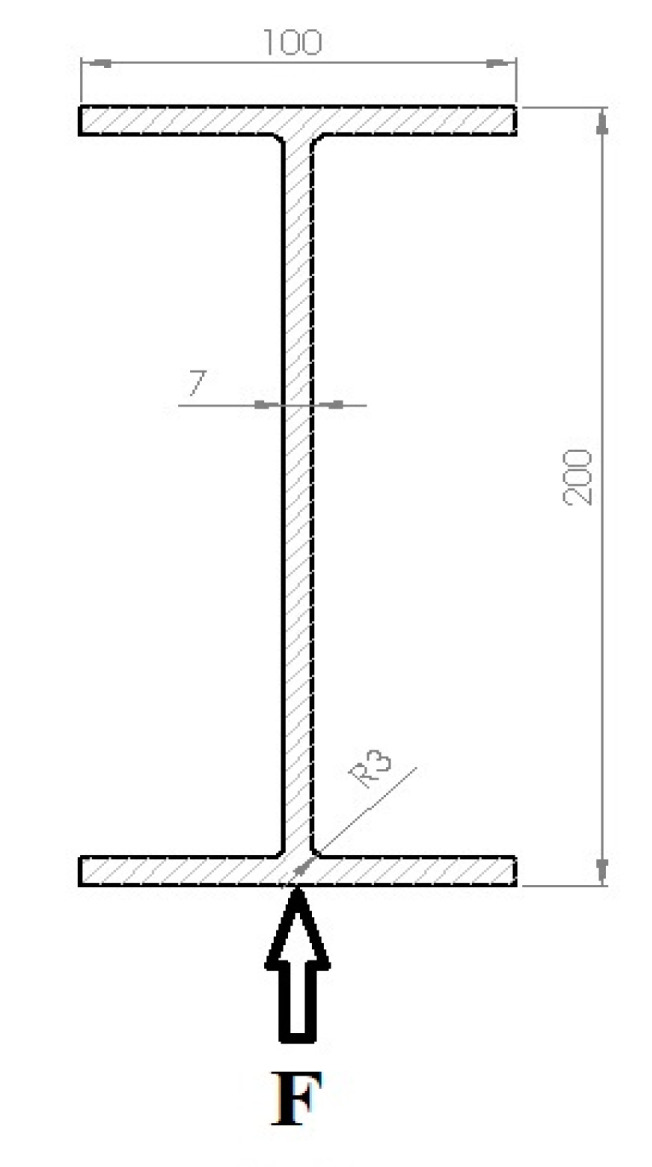
Crane’s girder cross-section, where: F is tested plane.

**Figure 4:**
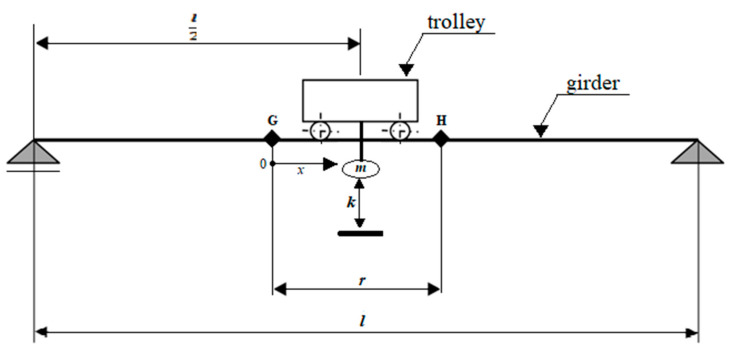
Experimental setup.

Research assumptions:

The experiments were carried out on the double-girder overhead traveling crane with hoisting capacity ***m*** = 1000 kg, a span of the girders ***l*** = 8 m, and trolley wheelbase ***p*** = 1 m. Figure 2 and Figure 3 present the crane subjected to examine, and girder beam cross-section, respectively. The girder is made of S235JR structural non-alloy steel [22]. The beam, supported on both sides at its ends, was subjected to elastic deformation, giving an elastic force in its center of 1000 kg, resulting in deflection of the beam f = 12.17 mm. The loading conditions of the tested girder are shown in Figure 3 and Figure 4.

The *r* = 1.4 m length section between points G and H (Figure 4) of the crane’s girder bottom surface was selected for examination using (where G is the starting point and *x* is the direction of the measurement). Points G and H are appointed symmetrically to the center of the crane beam.

**Figure 5:**
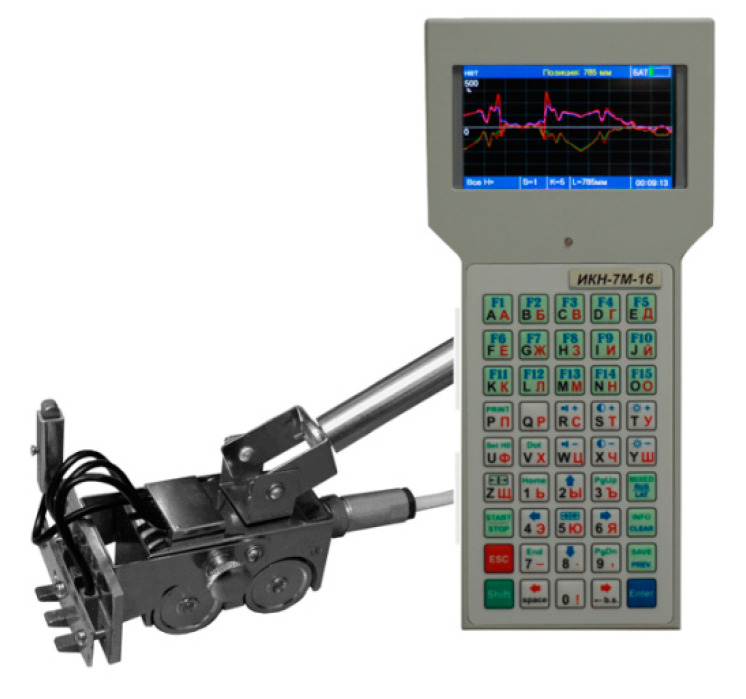
Tester of Stress Concentration TSC-4M-16 and scanning 4-channel sensor.

The SMFL signal was measured using the Tester Stress Concentration TSC-4M-16, which is equipped with a scanning device. The scanning device is a four-wheeled cart with the flux-gate transducers, and the encoder for sensing position of the cart moved along a measuring element (Figure 5). The magnetometers installed in the scanning device allow to measure *Hx* and *Hy* distribution of the self-magnetic leakage field signal along the surface of an inspected structure.

The measurement device allows to measure the magnetic field intensity in the range ±2000 A/m. The basic relative error of *H_p_* measurement is 5%, and the sensor movement accuracy is 0.9 mm. The magnetometer TSC-4M-16 is equipped with the software for data acquisition and analysis.

The measurements of the magnetic field strength in the designated spar section were carried out according to our own test plan.

During the inspection, the trolley with a winch was set in the middle of the crane’s girder. The load was placed in the center of the trolley. Figure 4 presents the dimensions and position of the trolley with a load during the experiments. The crane’s girder, where: ***l*** is crane span,$\frac{l}{2}$ is the distance of the suspended load from the beginning of the girder, ***m*** is load suspended on a rope, ***k*** is load-lifting height measured from the ground, ***r*** is the examined section between points G and H of the girder. In order to eliminate interference magnetic field strength, the tests were performed with the driving and lifting mechanisms turned off.

All measurements of the magnetic field strength were carried out in the same conditions:

- the trolley with the load was placed in the middle of the spar span,
- constant ambient temperature *T* = 22 °C,
- during the measurement, all devices disturbing the magnetic field are turned off,
- the same section of the spar’s lower surface was always tested.

The research was divided into two stages.

During the first stage, the intensity of the residual magnetic field in the spar loaded for 4 h was tested to the following scenario repeated every 24 h for 4 days, the results are presented in Table 1

- measurement variant A—first measurement with the crane load ***m*** = 0 kg, ***k*** *=* 0 cm, ***t*** = 0 s,
- measurement variant B—the experiment carried out immediately after the load was applied, ***m*** = 1000 kg, ***k*** *=* 10 cm, *t* = 0 s,
- measurement variant C—the experiment for the crane maximum load ***m*** = 1000 kg, ***k*** *=* 10 cm, ***t*** = 14,400 s (4 h) carried out four hours after variant B, performed after 4 h of loading of the crane girders with a load of ***m*** = 1000 kg,
- measurement variant D—immediately after the measurements in variant C, the load is removed from the girder, ***m*** = 0 kg, ***k*** = 0 cm, ***t*** = 0 s.

During the second stage of the research, measurements of the magnetic field strength were carried out after 10 loads of 1000 kg for 5 s:

- measurement variant E—The first measurements were made according to the measurement variant A, where *m* = 0 kg, *t* = 0 s, in Table 2, these measurements were called the measurement cycle 0. Then, the load *m* = 1000 kg was raised to ***k*** = 10 cm, after ***t*** = 5 s the load was abandoned (***m*** = 0 kg, ***t*** = 0 s). This operation was repeated 10 times. The movement mechanisms were turned off, then the magnetic field strength was measured (measurement cycle 10). Six cycles were performed, after each, the magnetic field intensity in a given beam section was examined.

Four repeated measurements were carried out for each case A, B, A, B, C, D and E.

## 3. Results and Discussion

The results of the measurements magnetic field strength were analyzed. During the analysis of the measurements, the focus was on the graphs based on the value of the magnetic field strength at a given section of the tested object. The results of measurement experiments carried out for each case A, B, C, D, and E are presented in Table 1 in the form of maximum, minimum, and average values of tangential *Hx* and normal *Hy* component of SMFL are listed as determined as the mean values of four measurements repeated for each case. Maximum, minimum, and average values of magnetic field strength are listed as *Hx* and *Hy* in Table 1.

**Figure 6:**
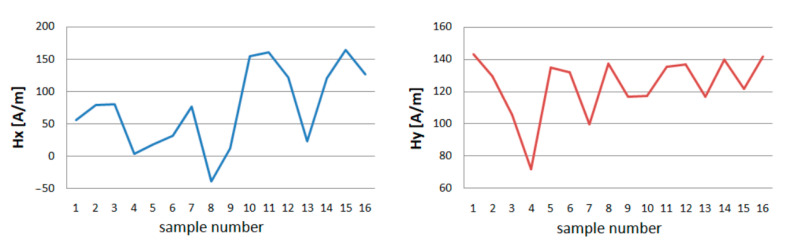
Minimum of tangential *Hx* and normal component *Hy* after repeatedly applying variants A, B, C, D, for 4 days long.

Graphs were made for *Hx* min and max and *Hy* min and max to compare the maximum, and minimum values of the magnetic field strength in the tested section of the crane girder during the experiment. These graphs are shown in Figure 6.

The minimal values of the tangential component ***x*** the strength of the magnetic field in the tested girder, after raising the load, reach higher values. With the passage of time under load, *Hx_min_* values are increasing. After unloading the crane, the magnetic field strength in the tested section of the *Hx_min_* girder decreases.

**Figure 7:**
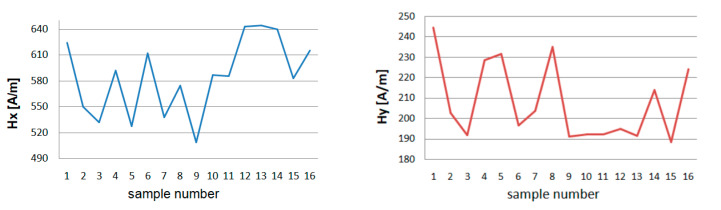
Maximum of tangential *Hx* and normal component *Hy* after repeatedly applying variants A, B, C, D, for 4 days long.

The lowest *Hx* value is obtained after several hours of relaxing the crane girder (Figure 7). The minimum normal component y of the magnetic field strength for each loading and unloading of the girder reacts with a change in value. The more measurement days, the smaller the range of change.

The same happens with max *Hx* and *Hy* values. Each operation related to the load causes a change in the value of the magnetic field strength for both components.

The maximum values of the magnetic field strength in the tested spar cross-section, after loading with the mass *m* = 1000 kg, decrease their value. After the load time *t* = 4 h, the measurement shows a decrease in the *H_max_* value for the *x* and *y* components. However, after removing the load where *m* = 0, *H_max_* reaches higher values.

**Figure 8:**
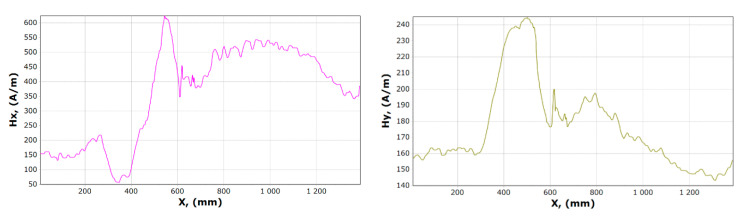
Graphs of tangential *Hx* and normal *Hy* component of the magnetic field, for sample 1.

**Figure 9:**
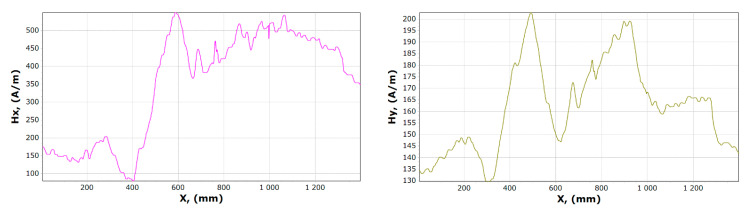
Graphs of tangential *Hx* and normal *Hy* component of the magnetic field, for sample 2.

**Figure 10:**
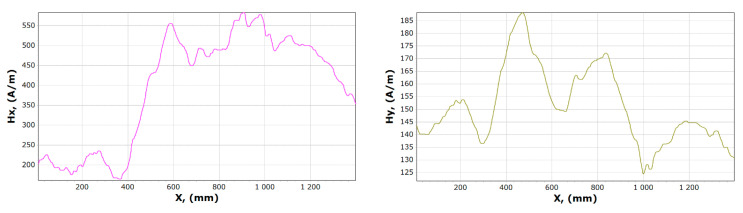
Graphs of tangential *Hx* and normal *Hy* component of magnetic field, for sample 15.

**Figure 11:**
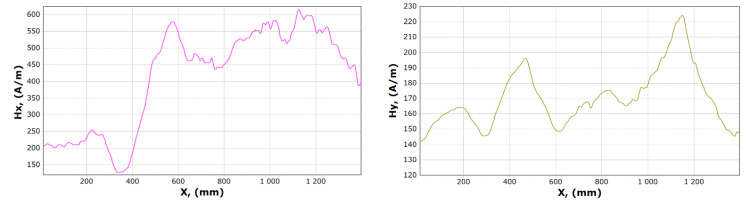
Graphs of tangential *Hx* and normal *Hy* component of the magnetic field, for sample 16.

Figure 8, Figure 9, Figure 10 and Figure 11 show graphs of tangential *Hx* and normal *Hy* component of the magnetic field, for samples 1, 2 and 15, 16. On their basis, it is possible to notice the characteristics of changes in magnetic field strength waveforms *Hx* and *Hy* at the beginning of the experiment, then immediately after the first loading of the device, and before the last unloading and after the whole experiment.

After comparing the charts of the *Hx* and *Hy* components, we notice slight differences in the course of the chart for *Hx*. The diagrams, regardless of the load and its length, are similar due to the curve. For *x* in the range 340–370 mm for unloaded girder, *Hx_min_* is about 50 A/m. After the last experiment, unloading the girder, this value is about 120 A/m. Whereas *Hx_max_* is in the range 550–600 mm is reduced from 620 to 570 A/m. The *Hy* changes its max and min and the course of the curve during each experiment. The graph shows tendencies to large changes in magnetic field strength for *Hy*. It can be stated that the magnetic field in the element subjected to stress reduces its amplitude vibration.

The next stage of the research included measurement variant E. The influence of dynamic loads on the intensity of the girder’s own magnetic field on the section determined in accordance with Figure 3 was examined.

**Figure 12:**
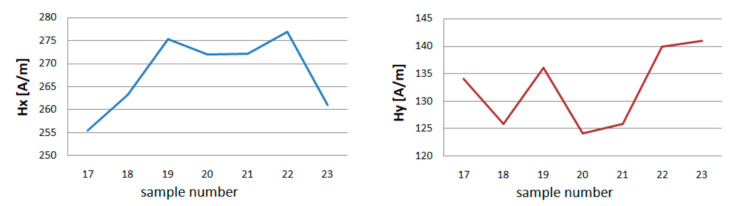
Minimum of tangential component *Hx* and *Hy* for experiment E.

**Figure 13:**
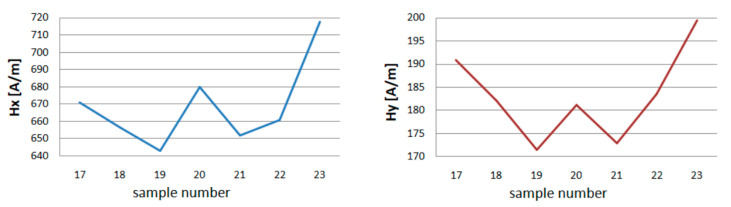
Maximum of tangential component *Hx* and *Hy* for experiment E.

After unloading the device magnetometric measurements were carried out. A total of 6 series of 10 loads and unloading of the girder were made. The measurement results for one magnetometer are presented in Table 2. The maximum, minimum, and average values of *Hx* and *Hy* are shown in Figure 12 and Figure 13.

After the first series of short loads on the crane girder, a change in the value of the magnetic field in the measured plane of the beam is noticeable. The minimum *Hx* values increase from 255 to 275 A/m, while the *Hy* decreases from 134 to 126 A/m. The second series of loads causes a slight decrease in the value of *H* for *x*. For *y*, *H_min_* achieves a value close to the measurement before the beam is loaded. The results of measurements of successive series show the independence of the magnetic field strength from the number of series made. The maximum values of *H* for *x* and *y* show a similar graph. After the first 10 loads, *H_max_* decreases, the next series increases the level of the magnetic field in the beam plane being tested for both components. In the third series, you can see a drop in value. After the last 6 trial, *H_max_* reaches the highest values *Hx_max_* = 719 A/m and *Hy_max_* = 200 A/m.

**Figure 14:**
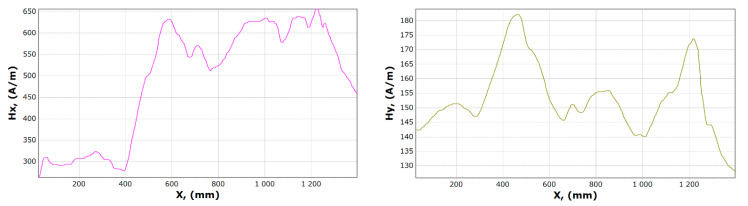
Graphs of tangential *Hx* and normal *Hy* component of the magnetic field, for sample 17.

**Figure 15:**
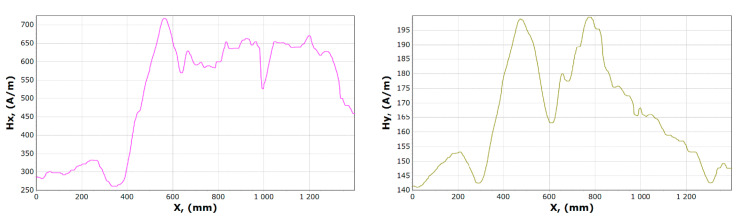
Graphs of tangential *Hx* and normal *Hy* component of the magnetic field, for sample 23.

Examples of magnetometric charts made on the basis of the first measurement taken without a girder load, and the last one is shown in Figure 14 and Figure 15.

The curve illustrating the *x* component before and after the experiment involving a similar course, when the curve illustrating the component *Hy* contains a significantly different character. The minimum *Hx* reaches in the range of 350–400 mm while the maximum in the range 550–600 mm. In the case of the *y* component, it is impossible to clearly determine the fixed place where it reaches its maximum and minimum.

## 4. Conclusions

The paper addresses the problem of the crane structure inspection by applying the MMM method.

The crane girder was the subject of inspections of the effect of load on changes in tangential and normal components of the magnetic field using MMM. When analyzing the results of measurement experiments, it can be seen that with each load change, the magnetic field strength changes. These changes are visible in Figure 5 and Figure 6. A similar situation occurs when the load is applied dynamically. After each series of measurements, the change in *Hx* and *Hy* measurements is visible (Figure 11 and Figure 12).

Based on the research, it can be concluded that short-range, high-value forces act between atoms of a given material with elastic properties. During elastic deformation of such material, interatomic distances and angles between atomic bonds change. As a result, it is associated with large energy changes because of the energy of elasticity increases. Elastic deformations are small and these small deformations are accompanied by relatively high forces. After the force causing the elastic deformation disappears, the material returns to its previous dimensions, the material atoms will take their previous positions again.

Microplastic deformations by dislocation development may cause changes in the magnetic flux leakage. However, according to our best knowledge, the relation between the microplastic deformation and magnetic flux leakage is not reported in the literature.

The tests may support the safety assessment of engineering structures subjected to loading. The knowledge of the influence of the load on the level of the magnetic field intensity allows us to predict the degradation time of the device structure. The use of the MMM method for monitoring can be helpful in the early detection of structural damage. This will result in increased work safety.

The next stage of the research will be to determine the intensity of the magnetic field during beam loads causing plastic deformation. These works are aimed at obtaining as much information as possible about the behavior of the magnetic field strength in the girders of the gantries working under load. Further research may be directed towards the challenging problem to obtain the damage indicator parameter and predict the absolute fatigue life of a crane’s structure. It requires analysis of magnetic flux leakage behavior under various load conditions during elastic and plastic deformations. Future work may consider to carry out more experiments, to collect more data and use data driven techniques to identify the influence of elastic load and plastic deformation on the magnetic flux leakage signal and their coupling effects.

## Figures and Tables

**Table 1:** Minimum, maximum, and average values of tangential and normal component of the magnetic signals for experiments A–D.

Measurement Day	A Sample	Measuremet Variant	*Hx_min_*	*Hx_max_*	*Hx_avg_*	*Hy_min_*	*Hy_max_*	*Hy_avg_*
1	1	A	56.6	624.3	360.7	143.4	244.5	176.2
	2	B	79.3	549.8	351.1	129.6	202.8	161.1
	3	C	81	532	334.2	105.6	191.9	137.9
	4	D	3.3	592	331.8	71.8	228.5	173.2
2	5	A	17.8	527.6	306.2	134.8	231.5	166.9
	6	B	32	612	197.9	131.8	196.6	16.3
	7	C	76.6	537.6	158.2	99.9	203.8	141.2
	8	D	−38.9	574.3	205.7	137.3	234.9	172.2
3	9	A	12.1	508.8	318.4	117	191.1	142.2
	10	B	154.4	586.5	391	117.1	192.1	160.5
	11	C	161	590	390.2	135.4	192.1	156.9
	12	D	122.1	643.1	4115	136.6	195	156.4
4	13	A	23.3	644.3	409.9	117	191.5	152.9
	14	B	121	639.8	4184	139.6	213.8	175.6
	15	C	164.4	583.1	397.5	121.6	188.3	150.2
	16	D	126.6	615.4	415.6	141.6	224.1	170

**Table 2:** Minimum, maximum, and average values of tangential and normal component of the magnetic signals for experiments E.

A Sample	Load Cycle	*Hx_min_*	*Hx_max_*	*Hx_avg_*	*Hy_min_*	*Hy_max_*	*Hy_avg_*
17	0	255.4	670.9	534.7	134.1	190.9	165
18	10	263.3	656.5	486.8	125.9	182.1	151.8
19	20	275.4	643.1	487.5	136.1	171.4	156.4
20	30	272	679.8	496.3	124.1	181.1	157.4
21	40	272.1	652	487.2	125.9	172.9	152.6
22	50	276.9	660.9	508.6	139.9	183.5	162.4
23	60	261	717.5	514	141	199.4	165.1

## References

[1] Bi Z., Kong L. (2018). The research on force-magnetic effect of high-speed train based on metal magnetic memory method. Proc. Chin. Intell. Syst. Conf..

[2] Hu Z., Fan J., Wu S., Dai H., Liu S. (2018). Characteristics of Metal Magnetic Memory Testing of 35CrMo Steel during Fatigue Loading. Metals.

[3] Ariffin A., Ahmad I M. (2015). Detection of cracked position due to cyclic loading for ferromagnetic materials based on magnetic memory method. J. Technol..

[4] Roskosz M., Bieniek M. (2012). Evaluation of residual stress in ferromagnetic steels based on residual magnetic field measurements. NDT E Int..

[5] Dubov A., Kolokolnikov S. (2013). The metal magnetic memory method application for online monitoring of damage development in steel pipes and welded joints specimens. Weld. World.

[6] Kosoń-Schab A., Szpytko J. (2019). Magnetic metal memory in the assessment of the technical condition of crane girders for the needs of safety. J. Konbin.

[7] Kosoń-Schab A., Smoczek J., Szpytko J. (2019). Magnetic memory inspection of an overhead crane girder—Experimental verification. J. Kones.

[8] Moonesan M., Kashefi M. (2018). Effect of sample initial magnetic field on the metal magnetic memory NDT result. J. Magn. Magn. Mater..

[9] Chongchong L., Lihong D., Haidou W., Guolu L., Binshi X. (2016). Metal magnetic memory technique used to predict the fatigue crack propagate on behavior of 0.45% C steel. J. Magn. Magn. Mater..

[10] Kolokolnikov S., Dubov A.A., Steklov O. (2016). Assessment of welded joints stress–strain state in homogeneity before and after post weld heat treatment based on the metal magnetic memory method. Weld. World.

[11] Dubov A.A. Principle features of metal magnetic memory method and inspection tools as compared to known magnetic NDT methods. Proceedings of the 16th Annual Conference on Nondestructive Testing.

[12] Juraszek J. (2019). Residual magnetic field for identification of damage in steel wire rope. Arch. Min. Sci..

[13] Dubov A.A. Energy Diagnostics is a physical basis of the metal *magnetic* memory method. Proceedings of the 11th European Conference on Non-Destructive Testing.

[14] Huang S., Wang S. (2016). Metal Magnetic Memory Testing. New Technologies in Electromagnetic Non-Destructive Testing.

[15] Chen L., Que P.W., Jin T. (2005). A giant-magnetoresistance sensor for magnetic-flux-leakage nondestructive testing of a pipe line. Russ. J. Nondes. Test..

[16] Sonntag N., Skrotzki B., Stegemann R., Löwe P., Kreutzbruck M. (2018). The role of surface topography on deformation-induced magnetization under inhomogeneous elastic-plastic deformation. Materials.

[17] Dubov A.A. (2015). Detection of metallurgical and production defects in engineering components using metal magnetic memory. Metallurgist.

[18] Ren S.K., Ren X.Z. (2018). Studies on laws of stress-magnetization based on magnetic memory testing technique. J. Magn. Magn. Mater..

[19] Shui G.S., Li C.W., Yao K. (2015). Non-destructive evaluation of the damage of ferromagnetic steel using metal magnetic memory and nonlinear ultrasonic method. Int. J. Appl. Electromagn. Mech..

[20] Shi P., Jin K., Zheng X. (2017). A magneto mechanical model for the magnetic memory method. Int. J. Mech. Sci..

[21] Vlasov V.T., Dubov A.A. (2004). Physical Bases of the Metal Magnetic Memory Method.

[22] (2019). EN 10025-2: 2019 Hot Rolled Products of Structural Steels—Part 2: Technical Delivery Conditions for Non-Alloy Structural Steels.

